# Peripheral Alzheimer's Disease Biomarkers Are Related to Change in Subjective Memory in Older Women with Cardiovascular Risk Factors in a Trial of Yoga vs. Memory Training: Lien établi entre les biomarqueurs périphériques de la maladie d’Alzheimer et l’amélioration de la mémoire subjective chez les femmes âgées présentant des facteurs de risque cardiovasculaire dans le cadre d’un essai comparant le yoga à l’entraînement de la mémoire

**DOI:** 10.1177/07067437251343291

**Published:** 2025-05-30

**Authors:** Hanadi Ajam Oughli, Prabha Siddarth, Meachelle Lum, Lara Tang, Brandon Ito, Matthew Abikenari, Monica Cappelleti, Dharma S. Khalsa, Sarah Nguyen, Helen Lavretsky

**Affiliations:** 1 Department of Psychiatry & Biobehavioral Sciences, David Geffen School of Medicine at University of California, Los Angeles, CA, USA; 2 Department of Psychiatry, Jane and Terry Semel Institute for Neuroscience and Human Behavior, University of California, Los Angeles, CA, USA; 3 David Geffen School of Medicine, 8783 University of California Los Angeles, Los Angeles, CA, USA; 4 Department of Psychiatry, 12295 Weill Cornell Medical Center/New York Presbyterian Hospital, New York, NY, USA; 5 10624 Stanford School of Medicine, Stanford, CA USA; 6 UCLA Immunogenetics Center, Department of Pathology & Lab Medicine, David Geffen School of Medicine at UCLA, Los Angeles, CA, USA; 7 Alzheimer's Research and Prevention Foundation, Tuscon, AZ, USA

**Keywords:** yoga, memory training, Alzheimer's disease, peripheral biomarkers, mechanisms

## Abstract

**Objectives:**

Older women with cardiovascular risk factors and subjective memory complaints are at greater risk for Alzheimer’s disease (AD). We examined the changes in AD peripheral biomarkers, including phosphorylated-tau (p-tau), Aβ40, Aβ42, and Aβ42/40 ratio, in a randomized controlled trial of Kundalini yoga (KY) versus memory enhancement training (MET) in aging women at risk for AD.

**Methods:**

We recruited women (50+ years) with subjective memory complaints and high cardiovascular risk as defined by the ACC/AHA Guideline on the Assessment of Cardiovascular Risk. Participants were randomized into KY versus MET, each lasting for 12 weeks, with a 24-week follow-up. We obtained blood samples at baseline and week 24 and measured Aβ 40, Aβ 42, and p-Tau. Participants completed the Memory Functioning Questionnaire (MFQ) to assess subjective memory at baseline and follow-up.

**Results:**

A total of 79 patients (KY = 40; MET = 39) were randomized, and biomarker data were available for 56 participants (KY = 24; MET = 32) at baseline and the 24-week follow-up. There were no group differences in AD biomarkers at baseline or at 24-week follow-up, and there were no significant changes in AD biomarkers from baseline to 24-week follow-up. Higher baseline levels of Aβ40 and Aβ42 were significantly associated with an improvement in subjective memory (MFQ Frequency of Forgetting and Seriousness of Forgetting) at follow-up. There was no significant association of the Aβ42/40 ratio and p-tau with changes in subjective memory.

**Conclusions:**

Our findings indicate that peripheral Aβ40 and Aβ42 levels are associated with improvement in memory self-awareness, particularly the reported frequency and perceived severity of forgetting. These levels may serve as potential biomarkers, reflecting underlying biological effects that could be utilized in future assessments. Further research is needed to determine how to successfully utilize peripheral biomarkers and subjective memory complaints to identify at-risk populations.

**Plain Language Summary Title:**

Peripheral Alzheimer’s Disease biomarkers are related to change in subjective memory in older women in a trial of yoga vs. memory training.

## Plain Language Summary

This randomized controlled trial investigated whether Kundalini yoga (KY) or memory enhancement training (MET) influenced Alzheimer’s disease (AD) peripheral biomarkers in older women with subjective memory complaints and elevated cardiovascular risk. Over 24 weeks, no significant group differences or changes in biomarkers were observed. However, higher baseline levels of Aβ40 and Aβ42 were significantly associated with improvements in subjective memory. These findings suggest that Aβ40 and Aβ42 may serve as potential biomarkers of memory self-awareness, warranting further research for early identification of individuals at risk for AD.

## Résumé

**Objectifs:**

Les femmes âgées présentant des facteurs de risque cardiovasculaire et des symptômes liés à la mémoire subjective ont un risque accru de maladie d’Alzheimer (MA). Nous avons examiné les variations des biomarqueurs périphériques de la MA, y compris les taux de protéine tau phosphorylée (p-tau), d’Aβ40, d’Aβ42 ainsi que le rapport Aβ42/40, dans le cadre d’un essai contrôlé à répartition aléatoire comparant le yoga Kundalini (KY) à l’entraînement d’amélioration de la mémoire (MET) chez des femmes âgées à risque de MA.

**Méthodes:**

Nous avons recruté des femmes (âgées de 50 ans et plus) présentant des symptômes liés à la mémoire subjective et un risque cardiovasculaire élevé, comme défini par les lignes directrices sur l’évaluation du risque cardiovasculaire de l’ACC/AHA. Les participantes ont été réparties de façon aléatoire entre les groupes KY et MET, chaque intervention ayant une durée de 12 semaines avec un suivi à la semaine 24. Nous avons prélevé des échantillons de sang au début de l’essai et à la semaine 24 pour mesurer les taux d’Aβ40, d’Aβ42 et de p-tau. Les participantes ont rempli le questionnaire sur le fonctionnement de la mémoire (Memory Functioning Questionnaire – MFQ) afin d’évaluer la mémoire subjective au début de l’essai et au moment du suivi.

**Résultats:**

Au total, 79 patientes (KY = 40; MET = 39) ont été réparties de façon aléatoire. Des données sur les biomarqueurs étaient disponibles pour 56 participantes (KY = 24; MET = 32) au début de l’essai et au moment du suivi à la semaine 24. Il n’y avait aucune différence liée au groupe dans les biomarqueurs de la MA au début de l’essai ni au moment du suivi à la semaine 24. De plus, aucune variation importante des biomarqueurs de la MA n’a été observée entre le début de l’essai et le moment du suivi à la semaine 24. Des taux supérieurs d’Aβ40 et d’Aβ42 au début de l’essai ont été associés à une amélioration significative de la mémoire subjective (éléments relatifs à la fréquence et à la gravité des oublis du MFQ) au moment du suivi. Il n’y avait pas d’association significative entre le rapport Aβ42/40 et le taux de p-tau, et les variations de la mémoire subjective.

**Conclusions:**

Nos résultats indiquent que les taux d’Aβ40 et d’Aβ42 périphériques sont associés à une meilleure conscience de soi dans l’auto-évaluation de la mémoire, particulièrement en ce qui a trait à la fréquence d’oublis signalée et à la gravité des oublis perçue. Ces taux peuvent servir de biomarqueurs potentiels pour refléter les effets biologiques sous-jacents et ils pourraient être utilisés dans de futures évaluations. D’autres recherches sont nécessaires pour déterminer comment utiliser efficacement les biomarqueurs périphériques et les symptômes liés à la mémoire subjective pour cerner les populations à risque.

## Introduction

Alzheimer's disease (AD) is an increasingly widespread neurodegenerative disease with no current available cure. Nearly two-thirds of individuals living with AD in the United States are women.^1^ Several factors may be responsible for this sex discrepancy, including increased life span, greater genetic susceptibility, higher rates of depression, and menopause.^2,3^ Simultaneously, the association of cardiovascular disease with higher AD risk has been well-established in both men and women.^4–7^ Yet, women's cognitive abilities appear to decline roughly twice as fast as those of their male counterparts, particularly in those who have been diagnosed with mild cognitive impairment (MCI).^8,9^

Enhancing brain health and cognition in women at risk for AD can help delay or prevent the onset of AD.^10^ Traditional forms of aerobic exercise (e.g., running, cycling, swimming) have been shown to be protective and reduce the risk of AD.^11^ Higher levels of physical activity and exercise have been shown to improve cognitive scores amongst women, even when limited to later in life.^12^ Furthermore, mind-body therapies, such as yoga, show beneficial effects on cognitive affective and physical function in older adults.^13^ Yoga training may offer neuroprotective effects through preventing neurodegenerative changes and cognitive decline in older adults with MCI.^14^ Prior research by our group has shown beneficial effects of Kundalini yoga (KY) on cognitive function, including executive function, neuroplasticity, and neurochemistry in older adults with MCI.^15–17^ KY uniquely altered aging-associated signatures, including interferon gamma and other psycho-neuro-immune pathways, thus suggesting clinical and biological benefits.^16,18^ KY further prevents gray matter atrophy in several cortices, including the left pre-frontal cortex, while increasing telomerase activity, brain metabolism, and decreasing NF-kappa-B-related (NF-kB) transcription of inflammatory cytokines.^19–21^

Peripheral blood biomarkers associated with AD can be identified before the formal diagnosis.^22,23^ Blood biomarkers correlate with changes in cognition and brain atrophy and could be used to inform treatment implementation and management in clinical practice.^24^ The accumulation of amyloid beta (Aβ) peptides, which are often found in extracellular plaques and intracellular neurofibrillary tangles that compromise the tau protein, is the defining criterion of AD. Quantifying specific plasma proteins such as Aβ40, Aβ42, Aβ42/40, and phosphorylated tau protein (p-tau) has been shown to provide a predictive value for the progression of MCI and AD.^25,26^

Both yoga and memory training can be beneficial in improving cognitive function and reducing the risk of AD in at-risk populations. It remains unclear whether yoga can affect peripheral AD biomarkers, thereby reducing the risk of disease progression. In this context, we conducted an exploratory analysis where we investigated the effects of yoga or memory training on peripheral biomarkers of AD (Aβ 40, Aβ 42, Aβ 42/40 ratio and p-tau) over time. We hypothesized that KY would improve the biomarkers over time (i.e., increase Aβ 42 and Aβ 42/40 ratio). We further examine the relationship of these biomarkers to subjective memory.

## Methods

### Parent Study

We conducted a randomized, controlled trial to assess the efficacy of KY training compared to memory enhancement training (MET) on mood and cognitive functioning in a group of older women with cerebrovascular risk factors and subjective memory complaints.^16^ The study and its methodology were described elsewhere in detail, and a brief description of the methods is presented in this manuscript.^16^ All study procedures were approved by the University of California Los Angeles’ (UCLA's) Institutional Review Board (NCT03503669).

We recruited older women aged 50+ years with subjective memory complaints and high cardio- and cerebro-vascular risk as defined by the 2013 ACC/AHA Guideline on the Assessment of Cardiovascular Risk. The primary outcomes of interest were changes in (1) cognitive domain scores, such as delayed recall and executive functioning, at 24 weeks as compared to baseline and (2) subjective memory scores at 12 and 24 weeks as compared to baseline. The secondary outcomes include changes in clinical measures such as depression, anxiety, perceived stress, resilience, and health-related quality of life. Additionally, a comprehensive cytokine/chemokine assay and analysis were conducted.

We found that at 24 weeks follow-up, KY was associated with a significantly large effect size improvement in subjective cognitive complaints but a significant decline in delayed recall compared to MET. Neither intervention resulted in changes in anxiety, depression, perceived stress, or resilience. Additionally, KY participants demonstrated reversal of aging-associated gene expression signatures and MET, but not KY participants, demonstrated increased levels of aging-associated chemokine exotoxin-1.

### Participants

We recruited older women with subjective memory complaints and reported decline in memory ability who were included in our study. Subjective memory complaints were characterized as the subjective experience of declining memory abilities, despite a normal range of memory function based on neuropsychological measures. Participants required to exhibit the following criteria: (1) self- reported memory difficulties occurring within the past six months; (2) a frequency of memory problems that occur at least once weekly; (3) ability to give an example in which memory difficulties occur in daily life; (4) one's belief that their memory capacity has declined as compared to 5–10 years ago; (5) concerns and worries about memory difficulties.

Participants were recruited from the UCLA's women's health, cardiology clinics. All participants provided informed consent.

All participants were at high cardio- and cerebro-vascular risk, defined by at least one of the following criteria: (1) a 7.5 percentile risk or higher based on the ASCVD risk calculator; (2) myocardial infarction more than 6 months ago; (3) diabetes diagnosis; (4) taking a medication for blood pressure; (5) blood pressure ≥ 140/90 mmHg; (6) taking medication for hyperlipidemia; (7) low density lipoprotein (LDL) ≥ 160 mg/dL. Participants also needed to have sufficient English proficiency to comprehend the intervention materials and the capacity to provide informed consent.

We excluded participants if they had a prior history of psychiatric illness such as bipolar disorder, psychosis, alcohol or substance dependence, or a neurological disorder. Additionally, we excluded older women who had clinically significant depressive symptoms as indicated by a BDI score of >17, unstable medical condition or recent surgery (within 3 months), any disability preventing participation in MET or KY interventions (e.g., severe visual or hearing impairment), diagnosis of dementia or global cognitive deficits as noted by a Mini-Mental Health Examination score of 23 or below, currently taking psychoactive medication(those on a stable antidepressant and are not currently depressed were included), participation in a psychotherapy that involves cognitive training, current yoga practice (frequency of once/week or greater), or myocardial infarction within the past 6 months. Additionally, participants needed to be physically capable of participating in yoga and meditation interventions.

### Study Interventions

Participants were randomized to either the MET class for 60 min per week plus daily homework or the KY class for 60 min per week plus daily Kirtan Kriya (KK) homework.

#### Memory enhancement training intervention

Participants were informed that MET teaches specific memory strategies and has shown to improve memory in adults with mild cognitive impairment. The standard detailed protocol for MET program is founded on evidence-based techniques that use verbal and visual association strategies and practical strategies for memory compensation.^16^ The MET is a manualized program developed by researchers at the UCLA Longevity Center and includes components adapted from other effective memory training programs^16^ such as: (1) education about memory; (2) preliminary instruction in the basic elements of memory strategies (i.e., “pre-training”); (3) instruction in specific memory strategies; (4) home practice with logs to track activity; (5) addressing non-cognitive factors such as self-confidence, anxiety, and negative expectations; (6) small groups (i.e., 10 persons) and short 60 min sessions. The MET program involved a scripted curriculum for the memory trainer and a companion workbook for every participant.^16^ In each session, around 15 min were devoted to reviewing the homework and 45 min were devoted to learning and practicing techniques. Specific techniques such as visual associative learning strategies for learning faces and names, verbal associative methods such as the use of stories to remember lists, organizational strategies, forming good memory habits to recall where one places items and what one has done in the recent past (e.g., locking doors, turning off appliances), and how to remember future tasks (i.e., appointments) were also included.

#### KY intervention

The KY class included 6–10 participants and was scheduled for 60 min weekly which was conducted by a certified yoga instructor. The content and structure of the class was similar across the 12 weeks and included: (1) tuning in (5 min); (2) warm up (10 min); (3) pranayama (10 min); (4) kriya (20 min); (5) meditation (12 min); (6) shavasana (3 min). Participants were also asked to engage in 12 min of daily KK meditation, as previously described.^16^ The meditations were guided by standardized CDs distributed to participants and can be practiced at home. The meditation includes repetitive finger movements or mudras, in addition to chanting of the mantra “Saa, Taa, Naa, Maa,” meaning “Birth, Life, Death, and Rebirth,” first chanted aloud and subsequently in whisper then silently for a total of 11 min with 1 min allocated to “tuning in” at the beginning and the final deep breathing relaxation accompanied by the visualization of light. The aim of this protocol is to engage different senses concurrently such as visualization, vocalization, as well as motor and sensory stimulation.

The in-class attendance was tracked by staff members. A maximum of two missed classes was permitted for each participant. Additionally, completed KK homework sheets were presented to staff during the class. During the intervention, participants were instructed not to engage in other mind-body practices such as Tai Chi, Qi Gong, or yoga.

### Outcome Measures

#### Biomarkers

We obtained blood samples at baseline and week 24. Whole blood was centrifuged at 2000 rpm for 10 min, and plasma was immediately stored at −80°C. The Simoa Human Neurology 3-Plex A assay (Quanterix, Billerica, MA, USA) was applied on the automated Simoa HD-X Analyzer platform (Quanterix). The measurement range was 0–540 pg/ml for Aβ 40; 0–240 pg/ml for Aβ 42 and 0–400 pg/ml for p-Tau. Samples were diluted 1:4 as per manufacture's instruction.

#### Clinical measures

All clinical measures were also administered at baseline and week 24.

The Memory Functioning Questionnaire scale (MFQ) assesses subjective memory functioning and consists of 64 items rated on a seven-point scale, and provides four factor scores: (1) Factor 1 frequency of forgetting, consists of 33 items and includes ratings of how often forgetting occurs in 28 different situations and five ratings of general memory performance, (2) Factor 2, seriousness of forgetting, which consists of 18 items and includes ratings of memory failure from 18 specific situations, (3) Factor 3, retrospective functioning, consists of 5 items and includes ratings of changes in current memory ability relative to five time points earlier in life, (4) Factor 4, mnemonics usage, which consists of 8 items and includes ratings of frequency of mnemonics usage in eight different situations. Higher scores are indicative of better memory functioning (less forgetting incidents, fewer use of mnemonics). The factor structure remains consistent across age groups, and exhibits high internal consistency, with Cronbach's alpha values for the four factor scores ranging from 0.83 to 0.94.

In the parent clinical trial comparing KY to MET, the only significant improvements observed among the clinical measures were in the MFQ.^16^ We focused on two of the more commonly used factors and are considered to have high face validity,^27^ namely the MFQ Frequency of Forgetting and MFQ Seriousness of forgetting, which have been shown to more robustly reflect AD pathology than other MFQ components.^28^ The MFQ Frequency of Forgetting subscale is more sensitive to cognitive performance than other scales both cross-sectionally^29,30^ as well as longitudinally.^31^ Both interventions, KY and MET, significantly improved the factor score for Frequency of Forgetting at 24-week follow-up compared to baseline, and only KY participants demonstrated significant improvements in Seriousness of Forgetting. Further, both Frequency of Forgetting and Seriousness of Forgetting demonstrated significant associations with underlying gene expression signatures at baseline.

### Statistical Analysis

All data were inspected for outliers, homogeneity of variance and other assumptions to ensure their appropriateness for parametric statistical tests. Blood biomarkers, including Aβ 40, Aβ 42, Aβ 42/40 and p-tau were log-transformed for analyses. Intervention groups were compared using t-tests (continuous variables) or chi-squared tests (categorical variables) on all demographic and outcomes measures at baseline. Changes in blood biomarkers were analyzed using a mixed effects general linear model, including treatment group, time, and the interaction between time and treatment group, controlling for age. Significance of the interaction between time and intervention group was used to determine whether the groups differed in changes. Association of baseline levels of biomarkers with changes in subjective memory were examined using general linear models, with MFQ factor change as the dependent variable, and biomarker, treatment group and the interaction between biomarker and treatment group, controlling for age. All analyses were conducted using SAS 9.4 (SAS Institute, Cary, North Carolina).

## Results

**Figure 1.**
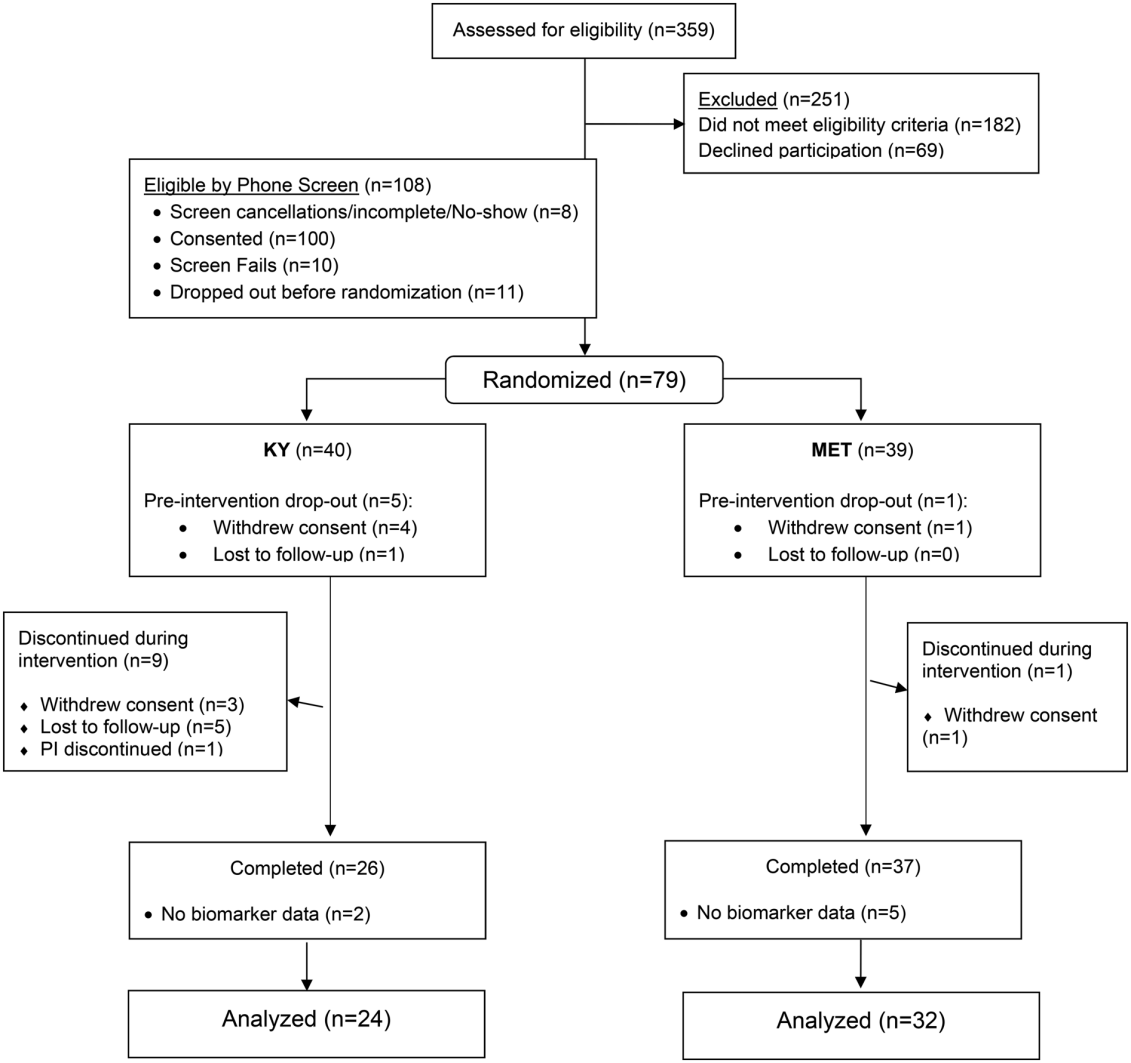
CONSORT diagram. KY = Kundalini yoga. MET = memory enhancement training.

A total of 79 participants were randomized to KY (*n* = 40) or MET (*n* = 39) (Figure 1). Descriptive and biomarker data were available for 56 participants (KY = 24; MET = 32). The mean age of our 56 participants at baseline was 65.2 (SD = 7.7) years, with mean educational level of 16.1 (SD = 1.9) years. Mean BMI was 27.6 (SD = 6.2), mean CVRF 9.4 (SD = 4.5), and mean MMSE was 28.5 (SD = 1.3). The baseline characteristics of the randomized participants are summarized in Table 1 by treatment group. At baseline, treatment groups did not differ significantly in age, race, years of education, body mass index (BMI) and cerebrovascular risk factors (CVRF).

**Table 1.** Baseline Characteristics of Participants in Study.

Characteristic	KY (*N* = 24) Mean (SD) or *N* (%)	MET (*N* = 32) Mean (SD) or *N* (%)
Age, years	62.9 (6.3)	67.5 (9.3)
Race		
White	16 (28.6%)	20 (35.7%)
Black	2 (3.6%)	4 (7.1%)
Asian	2 (3.6%)	5 (8.9%)
Hispanic	4 (7.1%)	1 (1.8%)
Other	0 (0%)	2 (3.57%)
Education, years	16.4 (1.7)	15.7 (2.0)
BMI	27.6 (5.2)	28.0 (6.8)
CVRF	8.5 (4.7)	10.1 (4.3)
MMSE	28.5 (1.8)	28.4 (1.1)
MFQ		
Frequency of Forgetting	4.3 (1.1)	4.6 (1.1)
Seriousness of Forgetting	3.8 (1.3)	4.6 (1.2)

Note: KY = Kundalini yoga and Kirtan Kriya + Meditation; MET = memory enhancement training; BMI = body mass index; CBR = cardiovascular risk factors; MMSE = Mini Mental State Examination; *MFQ *= Memory Functioning Questionnaire.

There were no within-group or between-group differences in AD biomarkers at 24-week follow-up. Changes in biomarkers were not significant within groups and did not differ between groups (Table 2).

**Table 2.** Biomarker Levels at Baseline and 24-Week Follow-up of Participants in Study.

Biomarker	KY (*N* = 24) Mean (SD)^a^	Within-group statistics^b^	MET (N = 32) Mean (SD)^a^	Within-group statistics^b^	Between-group statistics^b^
Aβ 40					
Baseline	62.4 (45.8)	*t*(54) = −0.13, *P* = 0.9	71.0 (52.4)	*t*(54) = −1.10, *P* = 0.3	*F*(1,54) = 0.38, *P* = 0.5
24-week	57.5 (41.6)		54.6 (37.0)		
Aβ 42					
Baseline	8.0 (5.7)	*t*(54) = −0.07, *P* = 0.9	9.2 (6.6)	*t*(54) = −1.03, *P* = 0.3	*F*(1,54) = 0.52, *P* = 0.5
24-week	7.9 (5.4)		7.0 (4.3)		
Aβ 42/40					
Baseline	0.15 (0.08)	*t*(54) = −0.28, *P* = 0.8	0.15 (0.07)	*t*(54) = 0.84, *P* = 0.4	*F*(1,54) = 0.58, *P* = 0.5
24-week	0.14 (0.05)		0.17 (0.12)		
p-tau					
Baseline	6.0 (4.7)	*t*(54) = −1.59, *P* = 0.1	4.8 (3.2)	*t*(54) = 0.54, *P* = 0.6	*F*(1,54) = 2.44, *P* = 0.1
24-week	4.1 (1.8)		6.2 (6.6)		

^a^ Note that all biomarker levels were log-transformed for analyses.

^b^ All statistics presented are obtained from mixed effects general linear models, including treatment group, time, and the interaction between time and treatment group as independent variables, controlling for age. Within-group statistics examine change of biomarker from baseline to 24-week follow-up and between-group statistics compare KY and MET on these changes.

**Figure 2.**
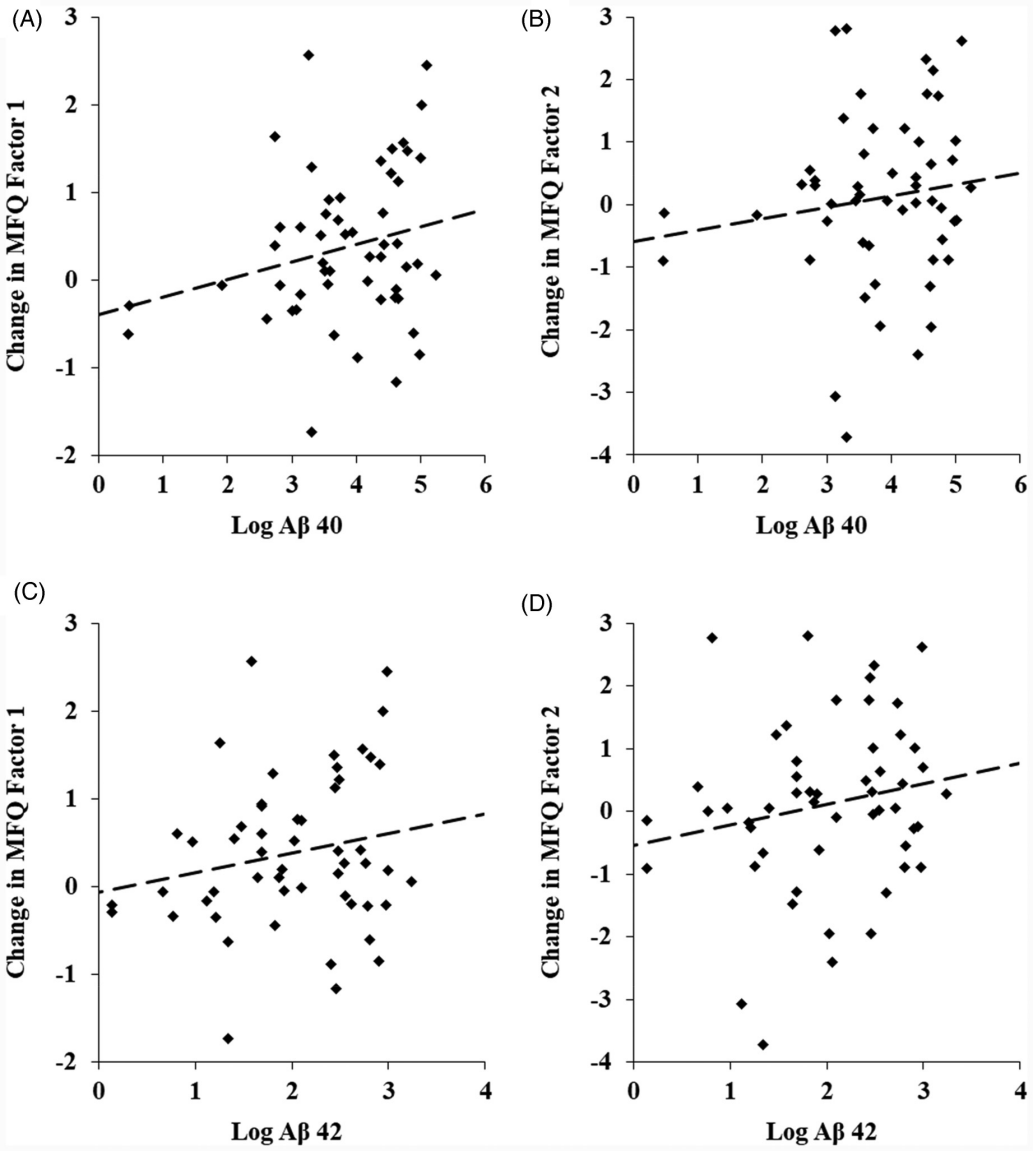
Associations of baseline levels of log Aβ 40 and log Aβ 42 with increase in MFQ Factor 1 (Frequency of Forgetting) and Factor 2 (Seriousness of Forgetting). (A) Baseline log Aβ 40 with change in Frequency of Forgetting (*b* = 0.29, SE = 0.12, *t* = 2.36, *P* = 0.02). (B) Baseline log Aβ 40 with change in Seriousness of Forgetting (*b* = 0.30, SE = 0.18, *t* = 1.68, *P* = 0.1). (C) Baseline log Aβ 42 with change in Frequency of Forgetting (*b* = 0.37, SE = 0.17, *t* = 2.26, *P* = 0.03). (D) Baseline log Aβ 42 with change in Seriousness of Forgetting (*b* = 0.51, SE = 0.24, *t* = 2.13, *P* = 0.04).

Examining the associations of baseline biomarker levels with changes in MFQ factor scores revealed no significant interaction with the treatment group, indicating that associations did not differ by intervention arm. Baseline levels of Aβ 40 were significantly associated with an increase in MFQ Frequency of Forgetting (*b* = 0.29, SE = 0.12, *t* = 2.36, *P* = 0.02; Figure 2). The association of Aβ 40 with change in MFQ Seriousness of Forgetting did not reach significance (*b* = 0.30, SE = 0.18, *t* = 1.68, *P* = 0.1). Additionally, baseline levels of Aβ 42 were significantly associated with increases in both MFQ factor scores (Frequency of Forgetting; *b* = 0.37, SE = 0.17, *t* = 2.26, *P* = 0.03; Seriousness of Forgetting: *b* = 0.51, SE = 0.24, *t* = 2.13, *P* = 0.04; Figure 2). There were no significant associations of Aβ 42/40 ratio and p-tau with changes in subjective memory.

## Discussion

This is the first study to examine the effects of KY yoga versus MET on AD peripheral biomarkers in older women with subjective memory complaints and cardiovascular risk factors. Overall, no significant differences were found in the changes of peripheral biomarker levels between the two intervention groups, KY yoga and MET. However, higher baseline levels of Aβ40 and Aβ42 were associated with increased scores on the MFQ Frequency of Forgetting and Seriousness of Forgetting at follow-up, suggesting better subjective memory.

To date, evidence linking subjective cognitive complaints with peripheral amyloid levels in older adults has been limited. Our finding that higher levels of peripheral Aβ40 and Aβ42 were associated with improvement in subjective memory suggests that these elevated Aβ levels may play a beneficial role in cognitive function. More specifically, higher plasma Aβ were associated with better perceived memory functioning and lower reported frequency of memory lapses. A study by Seppala et al. showed that higher levels of plasma Aβ42 were associated with cognitive stability over time.^32^ Meanwhile, the decreasing levels of plasma Aβ42 were indicators of recent cognitive decline and progression to AD.^32–35^ Similarly, a study by Innes et al. showed that improvements in memory functioning were positively correlated with increased plasma Aβ (Aβ 40, Aβ 42) levels, with frequency of forgetfulness showing the strongest association. Increases in Aβ levels were also positively associated with improvements in mood, perceived stress, psychological well-being, and multiple domains of quality of life.^36^ Aβ 40 has been shown to inhibit Aβ deposition and can interfere with Aβ 42 aggregation and could therefore be protective against amyloid pathology.^37^ Furthermore, recent studies noted that certain interventions which can increase plasma Aβ levels, e.g., antidiabetic medications, may also enhance cognition. An RCT of intranasal insulin in adults with MCI demonstrated significant increases in plasma Aβ40 levels, which were accompanied by improvements in memory and attention, all of which were notably greater than those receiving placebo. Likewise, another RCT showed that rosiglitazone, an insulin-sensitizing drug, led to improvement in delayed recall and enhanced selective attention in adults with MCI as compared to placebo. Plasma Aβ levels at 6 months remained stable from baseline in the rosiglitazone group, whereas they decreased in the placebo group, showing progressive decline. Thus, anti-diabetic medications and practices, such as music listening may alter plasma Aβ levels and biomarker increases, thereby increasing cognitive outcomes such as attention, memory, and functional status^36^ and could be used in the future.

The association between subjective cognition and Aβ levels in older adults with cognitive complaints can be measure-specific, with certain self-report scales reliably correlating with Aβ depositions/AD biomarkers. A study by Sntiz et al.^38^ found that cognitively normal older adults who exhibited worse subjective cognition, as indicated by their performance on the MFQ General Frequency of Forgetting factor, had significantly higher brain Aβ deposition as shown using carbon 11-labelled Pittsburgh Compound B Positron Emission Tomography (PiB PET) scans. Similarly, Amariglio et al. showed that the MFQ General Frequency of Forgetting factor and not the other three MFQ factors exhibited a significant association with brain Aβ deposition.^39^ This same MFQ factor pattern of association was demonstrated by Merrill et al. who showed that memory self-awareness, particularly the reported frequency of forgetting, was associated with greater medial temporal, parietal, frontal, and PET 2-(1-{6-[(2-[F-18]fluoroethyl)(methyl)amino]-2-naphthyl}ethylidene)malononitrile (FDDNP) uptake, thereby reflecting the extent of cerebral amyloid and tau brain pathology.^28^ Our findings are consistent with prior studies showing that the MFQ General Frequency of Forgetting closely correlates with Aβ pathology; however, unlike previous studies that primarily focused on brain Aβ deposition, we used the MFQ General Frequency of Forgetting to assess its association with peripheral plasma Aβ levels. This distinction highlights the potential of using the MFQ General Frequency of Forgetting as a measure for future research on peripheral AD biomarkers, offering a novel perspective on its utility.

We did not find significant associations between the peripheral Aβ42/40 ratio and p-tau levels with changes in subjective memory. Previous studies have demonstrated that higher concentrations of p-tau are associated with cognitive decline and memory impairment.^34^ Yet p-tau begins to accumulate during the mild cognitive impairment stage, so it cannot be reliably used as a predictor of AD, only as a diagnosis.^34^ Additionally, lower plasma Aβ42/40 ratios have been linked to an increased risk of Alzheimer's disease incident and greater cognitive decline.^32^ Our negative findings could be attributed to our limited sample size as well as shorter follow-up times.

There are several limitations in our study, including a small sample size and a relatively homogeneous sample in terms of educational level. Lower levels of education are associated with an increased risk of AD, and a highly educated sample may not be characteristic of the general population. Additionally, the intervention duration may not be sufficient to detect differences in peripheral biomarker changes, thus, longer interventions may be more beneficial. Further, lack of a “usual care” arm prevents estimation of age-related changes in AD biomarkers within the time of the follow-up. Finally, mixed models produce unbiased estimates when observations are missing at random; thus, we cannot rule out potential effects from data that are not missing at random.

## Conclusion

In conclusion, although we did not observe any differences in peripheral Aβ40 and Aβ42 levels between the KY and MET interventions, we did find a positive association between elevated Aβ40 and Aβ42 levels and improved subjective cognition, thereby suggesting that these markers can play a protective role against cognitive decline. The MFQ Frequency of Forgetting, in particular, can be used as a useful tool to further study in relation to preclinical AD biomarkers. Further research is needed to evaluate the relationship between changes in plasma Aβ biomarkers and subjective cognitive complaints in older adults, and to explore the role of multi-modal interventions to influence brain health, cognition and biomarkers.
